# Coronavirus Genomics and Bioinformatics Analysis

**DOI:** 10.3390/v2081803

**Published:** 2010-08-24

**Authors:** Patrick C. Y. Woo, Yi Huang, Susanna K. P. Lau, Kwok-Yung Yuen

**Affiliations:** 1 State Key Laboratory of Emerging Infectious Diseases, The University of Hong Kong, Hong Kong; China; E-Mail: kyyuen@hkucc.hku.hk; 2 Research Centre of Infection and Immunology, The University of Hong Kong, Hong Kong; China; 3 Carol Yu Centre of Infection, The University of Hong Kong, Hong Kong; China; 4 Department of Microbiology, The University of Hong Kong, University Pathology Building, Queen Mary Hospital, Hong Kong; China; E-Mail: huangyil@hkucc.hku.hk

**Keywords:** coronavirus, genome, bioinformatics

## Abstract

The drastic increase in the number of coronaviruses discovered and coronavirus genomes being sequenced have given us an unprecedented opportunity to perform genomics and bioinformatics analysis on this family of viruses. Coronaviruses possess the largest genomes (26.4 to 31.7 kb) among all known RNA viruses, with G + C contents varying from 32% to 43%. Variable numbers of small ORFs are present between the various conserved genes (ORF1ab, spike, envelope, membrane and nucleocapsid) and downstream to nucleocapsid gene in different coronavirus lineages. Phylogenetically, three genera, *Alphacoronavirus*, *Betacoronavirus* and *Gammacoronavirus*, with *Betacoronavirus* consisting of subgroups A, B, C and D, exist. A fourth genus, *Deltacoronavirus*, which includes bulbul coronavirus HKU11, thrush coronavirus HKU12 and munia coronavirus HKU13, is emerging. Molecular clock analysis using various gene loci revealed that the time of most recent common ancestor of human/civet SARS related coronavirus to be 1999–2002, with estimated substitution rate of 4×10^−4^ to 2×10^−2^ substitutions per site per year. Recombination in coronaviruses was most notable between different strains of murine hepatitis virus (MHV), between different strains of infectious bronchitis virus, between MHV and bovine coronavirus, between feline coronavirus (FCoV) type I and canine coronavirus generating FCoV type II, and between the three genotypes of human coronavirus HKU1 (HCoV-HKU1). Codon usage bias in coronaviruses were observed, with HCoV-HKU1 showing the most extreme bias, and cytosine deamination and selection of CpG suppressed clones are the two major independent biological forces that shape such codon usage bias in coronaviruses.

## 1. Introduction

Traditionally, viruses were characterized and classified by culture, electron microscopy and serological studies. Using these phenotypic methods, coronaviruses were defined as enveloped viruses of 120–160 nm in diameter with a crown-like appearance. The name “coronavirus” is derived from the Greek *κορώνα*, meaning crown. Based on their antigenic relationships, coronaviruses were classified into three groups. Group 1 and 2 are composed of mammalian coronaviruses and group 3 avian coronaviruses. The invention of and advances in nucleic acid amplification technologies, automated DNA sequencing and bioinformatics tools in the recent two decades have revolutionized the characterization and classification of all kinds of infectious disease agents. Using molecular methods, coronaviruses are classified as positive-sense, single-stranded RNA viruses. Furthermore, the results of using phylogenetic methods for classification also supported the group boundaries of the traditional antigenic classification. Phylogenetic methods have also enabled the classification of SARS-related coronavirus (SARSr-CoV) as a subgroup of group 2, group 2b, coronavirus; as well as the discovery of group 2c, 2d, 3b and 3c coronaviruses [1–3]. Recently, the Coronavirus Study Group of the International Committee for Taxonomy of Viruses has proposed three genera, *Alphacoronavirus*, *Betacoronavirus* and *Gammacoronavirus*, to replace these three traditional groups of coronaviruses [4].

The first complete genome of coronavirus, mouse hepatitis virus (MHV), was sequenced more than 50 years after it was isolated. Before the SARS epidemic in 2003, there were less than 10 coronaviruses with complete genome sequences available. These include two human coronaviruses (HCoV-229E and HCoV-OC43), four other mammalian coronaviruses [MHV, bovine coronavirus (BCoV), transmissible gastroenteritis virus (TGEV), porcine epidemic diarrhea virus (PEDV)], and one avian coronavirus (IBV). The SARS epidemic that originated from southern China in 2003 has boosted interest in all areas of coronavirus research, most notably, coronavirus biodiversity and genomics [5–7]. After the SARS epidemic, up to April 2010, 15 novel coronaviruses were discovered with their complete genomes sequenced. Among these 15 previously unrecognized coronaviruses were two globally distributed human coronaviruses, human coronavirus NL63 (HCoV-NL63) and human coronavirus HKU1 (HCoV-HKU1) [8–10]; 10 other mammalian coronaviruses, SARS-related *Rhinolophus* bat coronavirus (SARSr-Rh-BatCoV), *Rhinolophus* bat coronavirus HKU2 (Rh-BatCoV HKU2), *Tylonycteris* bat coronavirus HKU4 (Ty-BatCoV HKU4), *Pipistrellus* bat coronavirus HKU5 (Pi-BatCoV HKU5), *Miniopterus* bat coronavirus HKU8 (Mi-BatCoV HKU8), *Rousettus* bat coronavirus HKU9 (Ro-BatCoV HKU9), *Scotophilus* bat coronavirus 512 (Sc-BatCoV 512), *Miniopterus* bat coronavirus 1A/B (Mi-BatCoV 1A/B), equine coronavirus (ECoV) and beluga whale coronavirus SW1 [3,6,11–15]; and three avian coronaviruses, bulbul coronavirus HKU11 (BuCoV HKU11), thrush coronavirus HKU12 (ThCoV HKU12) and munia coronavirus HKU13 (MunCoV HKU13) [2]. Most of these genomes were sequenced using the RNA extracted directly from the clinical specimens, such as nasopharyngeal aspirate or stool, as the template, while the viruses themselves were still non-cultivable [2,3,6,11–15]. This provided more accurate analysis of the *in situ* viral genomes avoiding mutational bias during *in vitro* viral replication. These sequence efforts have resulted in a marked increase in the number of coronavirus genomes and have given us an unprecedented opportunity to understand this family of virus at the genomic and *in silico* levels. These understandings have also led to generation of further hypotheses and experiments in the laboratory. In this article, we reviewed our current understanding on the genomics and bioinformatics analysis of coronaviruses. Details of the bioinformatics tools will not be discussed.

## 2. Genomics

**Figure 1.**
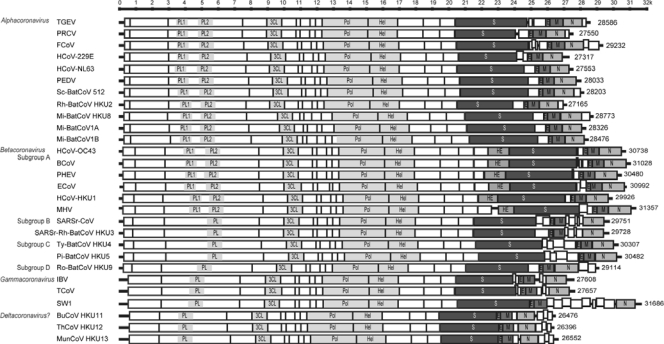
Genome organizations of members in different genera of the *Coronaviridae* family. PL1, papain-like protease 1; PL2, papain-like protease 2; PL, papain-like protease; 3CL, chymotrypsin-like protease; Pol, RNA-dependent RNA polymerase; Hel, helicase; HE, haemagglutinin esterase; S, spike; E, envelope; M, membrane; N, nucleocapsid. TGEV, porcine transmissible gastroenteritis virus (NC_002306); PRCV, porcine respiratory coronavirus (DQ811787); FCoV, feline coronavirus (NC_012937); HCoV-229E, human coronavirus 229E (NC_002645); HCoV-NL63, human coronavirus NL63 (NC_005831); PEDV, porcine epidemic diarrhea virus (NC_003436); Sc-BatCoV 512, *Scotophilus* bat coronavirus 512 (NC_009657); Rh-BatCoV-HKU2, *Rhinolophus* bat coronavirus HKU2 (NC_009988); Mi-BatCoV-HKU8, *Miniopterus* bat coronavirus HKU8 (NC_010438); Mi-BatCoV 1A, *Miniopterus* bat coronavirus 1A (NC_010437); Mi-BatCoV 1B, *Miniopterus* bat coronavirus 1B (NC_010436); HCoV-OC43, human coronavirus OC43 (NC_005147); BCoV, bovine coronavirus (NC_003045); PHEV, porcine hemagglutinating encephalomyelitis virus (NC_007732); HCoV-HKU1, human coronavirus HKU1 (NC_006577); MHV, mouse hepatitis virus (NC_006852); ECoV, equine coronavirus (NC_010327); SARSr-CoV, human SARS related coronavirus (NC_004718); SARSr-Rh-BatCoV HKU3, SARS-related *Rhinolophus* bat coronavirus HKU3 (NC_009694); Ty-BatCoV-HKU4, *Tylonycteris* bat coronavirus HKU4 (NC_009019); Pi-BatCoV-HKU5, *Pipistrellus* bat coronavirus HKU5 (NC_009020); Ro-BatCoV-HKU9, *Rousettus* bat coronavirus HKU9 (NC_009021); IBV, infectious bronchitis virus (NC_001451); TCoV, turkey coronavirus (NC_010800); SW1, beluga whale coronavirus (NC_010646); BuCoV HKU11, bulbul coronavirus HKU11 (FJ376620); ThCoV HKU12, thrush coronavirus HKU12 (NC_011549); MunCoV HKU13, munia coronavirus HKU13 (NC_011550).

Coronaviruses possess the largest genomes [26.4 kb (ThCoV HKU12) to 31.7 kb (SW1)] among all known RNA viruses (Figure 1) [2,13,16]. The large genome has given this family of virus extra plasticity in accommodating and modifying genes. The G + C contents of coronavirus genomes vary from 32% (HCoV-HKU1) to 43% (Pi-BatCoV HKU5 and MunCoV HKU13) (Table 1) [2,3,10]. Both the 5′ and 3′ ends of coronavirus genomes contain short untranslated regions. For the coding regions, the genome organizations of all coronaviruses are similar, with the characteristic gene order 5′-replicase ORF1ab, spike (S), envelope (E), membrane (M), nucleocapsid (N)-3′, although variable numbers of additional ORFs are present in each subgroup of coronavirus (Table 1, Figure 1). A transcription regulatory sequence (TRS) motif is present at the 3′ end of the leader sequence preceding most ORFs (Table 1). The TRS motifs are thought to be important for a “copy-choice” mechanism that mediates the unique random template switching during RNA replication, resulting in a high frequency of homologous RNA recombination in coronaviruses [17].

### 2.1. ORF1ab

ORF1ab of coronaviruses occupy about two thirds of their genomes. It encodes the replicase polyprotein and is translated from ORF1a (11826 to 13425 nt) and ORF1b (7983 to 8157 nt). In all coronaviruses, a slippery sequence (UUUAAAC), followed by sequences that form a putative pseudoknot structure, are present at the junction between ORF1a and ORF1b. Translation occurs by a -1 RNA-mediated ribosomal frameshift at the end of the slippery sequence. Instead of reading the transcript as UUUAAACGGG, it will be read as UUUAAACCGGG. The replicase polyprotein is cleaved by papain-like protease(s) (PL^pro^) and 3C-like protease (3CL^pro^), proteins encoded by ORF1ab of the coronavirus genome, at consensus cleavage sites, into 15 to 16 non-structural proteins (nsps) named nsp1, nsp2, nsp3, etc (Table 1). As the number of coronavirus genomes is expanding, novel cleavage sites have been discovered [3,18]. Some of these non-structural proteins encode proteins of essential functions, such as PL^pro^ (nsp3), 3CL^pro^ (nsp5), RNA-dependent RNA polymerase (Pol) (nsp12) and helicase (nsp13) (Figure 1). The genomes of all known members of *Alphacoronavirus* and *Betacoronavirus* subgroup A possess two PL^pro^ (PL1^pro^ and PL2^pro^), while those of all known members of *Betacoronavirus* subgroup B, C and D and *Gammacoronavirus* possess only one PL^pro^ (Table 1, Figure 1). The gene sequences that encode these conserved proteins are frequently used for phylogenetic analysis.

In addition to the nsps with essential functions, bioinformatics analysis of some other nsps revealed their putative functions. Downstream to PL^pro^ or PL1^pro^ in nsp3 is the X domain which contains putative ADP-ribose 1″-phosphatase (ADRP) activity [1]. In other microorganisms, such as *Saccharomyces cerevisiae* and other eukaryotes, ADRP and its functionally related enzyme cyclic nucleotide phosphodiesterase (CPDase), were important for tRNA processing [19]. ADP-ribose 1″,2″-cyclic phosphate (Appr>p) is produced as a result of tRNA splicing. Appr>p is in turn converted to ADP-ribose 1″-phosphate (Appr-1″p) by CPDase and Appr-″p is then further processed by ADRP. As for nsp13, nsp14 and nsp15, they possess a putative 3′-to-5′ exonuclease (ExoN) domain of the DEDD superfamily [1], a putative poly(U)-specific endoribonuclease (XendoU) domain, and a putative S-adenosylmethionine-dependent ribose 2′-O-methyltransferase (2′-O-MT) domain of the RrmJ family respectively [1]. ADRP, CPDase, ExoN, XendoU and 2′-O-MT are enzymes in RNA processing pathways. Contrary to the pre-tRNA splicing pathway that ADRP and CPDase belong to, ExoN, XendoU and 2′-O-MT are enzymes in a small nucleolar RNA processing and utilization pathway.

### 2.2. Haemagglutinin esterase

In all members of *Betacoronavirus* subgroup A, a haemagglutinin esterase (HE) gene, which encodes a glycoprotein with neuraminate O-acetyl-esterase activity and the active site FGDS, is present downstream to ORF1ab and upstream to S gene (Figure 1). The HE gene of coronavirus is believed to be acquired from influenza C virus, and is the most notable example of acquisition of new genes from non-coronavirus RNA donors by heterologous recombination [20]. The presence of HE genes exclusively in members of *Betacoronavirus* subgroup A, but not members of *Betacoronavirus* subgroup B, C and D suggested that the recombination had probably occurred in the ancestor of members of *Betacoronavirus* subgroup A, after diverging from the ancestor of other subgroups of *Betacoronavirus*.

### 2.3. Spike

The S proteins are responsible for the “spikes” present on the surface of coronaviruses and give this family of virus the characteristic crown-like appearance under electron microscopy. The S proteins are type I membrane glycoproteins with signal peptides. The S proteins are used for receptor binding and viral entry, and are the proteins with the most variable sequences in the coronavirus genomes. In some coronaviruses, the S proteins are cleaved into the S1 and S2 domains at consensus cleavage site (RRSRR of BCoV, RRSR of HCoV-OC43, RRKRR of HCoV-HKU1, RSRR of PHEV, RRADR of MHV, RRFRR of SDAV and RRFRR of IBV) (Table 1), with the sequences of the S1 domains much more variable than the S2 domains. In all coronaviruses, most of the S protein is exposed on the outside of the virus, with a short transmembrane domain at the C terminus, followed by a short cytoplasmic tail rich in cysteine residues. Two heptad repeats are present at the C termini of the extracellular parts of the S proteins. At the moment, no bioinformatics tool is available for accurate prediction of the receptor by analyzing the amino acid sequences of the S proteins of the corresponding coronaviruses.

### 2.4. Envelope and membrane

The E and M proteins are small transmembrane proteins associated with the envelope of all coronaviruses. In some coronaviruses, such as MHV and SDAV and possibly HCoV-HKU1, the translation of the E protein is cap-independent, via an internal ribosomal entry site. Although these two genes are conserved among all coronaviruses, they are not good targets for phylogenetic studies because of their short sequences.

### 2.5. Nucleocapsid

Similar to the conserved proteins encoded by ORF1ab, the N gene is also another common target for phylogenetic analysis. Due to its immunogenicity, it is also a common target for cloning and generation of recombinant proteins for serological assays.

### 2.6. Other small ORFS

Variable numbers of small ORFs are present between the various conserved genes in different lineages in the *Coronaviridae* family (Table 1, Figure 1). In some coronaviruses, small ORFs are present downstream to the N gene (Table 1, Figure 1). Most of these small ORFs are of unknown function. One exception is the small ORFs downstream to N in feline infectious peritonitis virus (FIPV) and TGEV, which are important for virulence and viral replication/assembly respectively [21–23]. Another notable exception is the 3a protein of SARSr-CoV, which forms a transmembrane homotetramer complex with ion channel function and modulates virus release [24]. For some of these small ORFs, such as ORF3a and ORF8 of SARSr-CoV genomes, their sequences are as highly variable as those of the S proteins. In particular, the most significant difference between human SARSr-CoV and civet SARSr-CoV genomes was a 29-bp deletion in the ORF8 of human SARSr-CoV [25].

## 3. Phylogeny

**Figure 2.**
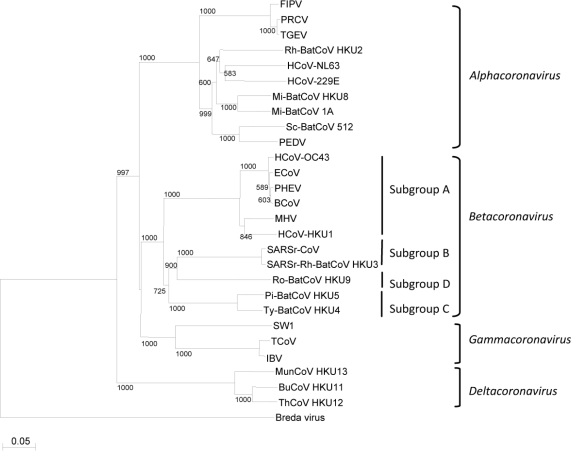
Phylogenetic analysis of RNA-dependent RNA polymerases (Pol) of coronaviruses with complete genome sequences available. The tree was constructed by the neighbor-joining method and rooted using Breda virus polyprotein (YP_337905). Bootstrap values were calculated from 1000 trees. 1118 amino acid positions in Pol were included. The scale bar indicates the estimated number of substitutions per 20 amino acids. All abbreviations for the coronaviruses were the same as those in Figure 1.

The first impression of the phylogenetic position of a strain or species of coronavirus is usually acquired by constructing a phylogenetic tree using a short fragment of a conserved gene, such as Pol or N. However, this can sometimes be misleading because the results of phylogenetic analysis using different genes or characters can be different. When SARSr-CoV was first discovered, it was proposed that it constituted a fourth group of coronavirus [26,27]. However, analyses of the amino-terminal domain of S of SARSr-CoV revealed that 19 out of the 20 cysteine residues were spatially conserved with those of the consensus sequence for *Betacoronavirus* [28]. On the other hand, only five of the cysteine residues were spatially conserved with those of the consensus sequences in *Alphacoronavirus* and *Gammacoronavirus* [28]. Furthermore, subsequent phylogenetic analysis using both complete genome sequence and proteomic approaches, it was concluded that SARSr-CoV is probably an early split-off from the *Betacoronavirus* lineage [1], and SARSr-CoV was subsequently classified as *Betacoronavirus* subgroup B and the historical *Betacoronavirus* as *Betacoronavirus* subgroup A. Therefore, the phylogenetic position of a coronavirus is best appreciated and confirmed by constructing phylogenetic trees using different genes in the coronavirus genome. The most commonly used genes along the coronavirus genome for phylogenetic studies include chymotrypsin-like protease, Pol (Figure 2), helicase, S and N, because these genes are present in all coronavirus genomes and are of significant length. The envelope and membrane genes, although present in all coronavirus genomes, are too short for phylogenetic studies. It is noteworthy that the cluster formed by the three novel avian coronaviruses BuCoV HKU11, ThCoV HKU12 and MunCoV HKU13, which was originated proposed as group 3c [2], might represent a new coronavirus genus provisionally designated *Deltacoronavirus* (Figure 2).

Using this approach of multiple gene phylogenetic studies, unique phylogeny of individual gene that may have biological significance may be discovered. During our phylogenetic study on Rh-BatCoV HKU2, another coronavirus that has was also found in the stool samples of Chinese horseshoe bats, its unique S protein phylogenetically distinct from the rest of the genome was discovered [15]. The S protein of Rh-BatCoV HKU2 is the shortest among S proteins of all coronaviruses and had less than 30% amino acid identities to those of all known coronaviruses, in contrast to other genes that showed higher amino acid identities to the corresponding genes in other members of *Alphacoronavirus*. When the S protein of Rh-BatCoV HKU2 is aligned with those of other members of *Alphacoronavirus*, many of the amino acid residues conserved among and specific to *Alphacoronavirus* were not found. Rather, the S protein of Rh-BatCoV HKU2 shares the two conserved regions of deletions both of 14 amino acids among members of *Betacoronavirus* in its C-terminus, suggesting that this segment of the S protein of Rh-BatCoV HKU2 may have co-evolved with the corresponding regions in *Betacoronavirus*. Most interestingly, a short peptide of 15 amino acids in the S protein of Rh-BatCoV HKU2 was found to be homologous to a corresponding peptide within the RBM in the S1 domain of SARSr-CoV. A similar peptide was also observed in SARSr-Rh-BatCoV, but not in any other known coronaviruses. These suggested that there is a common evolutionary origin in the S protein of SARSr-CoV, SARSr-Rh-BatCoV and Rh-BatCoV HKU2, and Rh-BatCoV HKU2 might have acquired its unique S protein from a yet unidentified coronavirus through recombination.

## 4. Evolutionary rate and divergence

In 1992, Sanchez *et al.* analyzed 13 enteric and respiratory TGEV related isolates and estimated the mutation rate of TGEV to be 7×10^−4^ nucleotide substitutions per site per year [29]. 1n 2005, using linear regression, maximum likelihood and Bayesian inference methods, Vijgen *et al.* estimated the rate of evolution in BCoV to be 4.3 (95% confidence internal 2.7 to 6.0) ×10^−4^ nucleotide substitutions per site per year [30]. The estimation of time of divergence was first extensively used in coronaviruses after the SARS epidemic for estimating the date of interspecies jumping of SARSr-CoV from civets to humans and that from BCoV to HCoV-OC43 [31,32]. Subsequently, when various novel human and animal coronaviruses were discovered, evolutionary rates and divergence time in the *Coronaviridae* family were estimated by various groups using different approaches [31,33–35]. Although Bayesian inference in BEAST is probably the most widely accepted approach and was used by most researchers, the use of different genes (ORF1ab, helicase, S and N genes) and datasets by different groups have resulted in considerable difference in the estimated history of coronaviruses. It was found that the S and N genes of PHEV, BCoV and HCoV-OC43 evolved at different rates, and the divergence time of the PHEV lineage and the HCoV-OC43 and BCoV lineage based on these two rates were 100 years different [31]. One group, using the helicase gene for analysis, has estimated the life history of coronaviruses to be as short as about 420 years [35].

Recently, we used the uncorrelated exponentially distributed relaxed clock model (UCED) in BEAST version 1.4 [36] to estimate the time of divergence of SARSr-CoV based on an alignment of a large set of SARSr-Rh-BatCoV ORF1 sequences collected over a period of five years. Under this model, the rates were allowed to vary at each branch drawn independently from an exponential distribution. Using this model and large dataset, the time of emergence of SARSr-CoV was at 1972, about 31 years before the SARS epidemic; that of SARSr-CoV in civet was at 1995, about eight years before the SARS epidemic; and the most recent common ancestor date of human and civet SARSr-CoV was estimated to be 2001.36, which was comparable to the dates estimated by other groups (Table 2) [37].

## 5. Recombination analysis

As a result of their unique random template switching during RNA replication, thought to be mediated by a “copy-choice” mechanism, coronaviruses have a high frequency of homologous RNA recombination [41,42]. Recombination in coronaviruses was first recognized between different strains of MHV and subsequently in other coronaviruses such as IBV, between MHV and BCoV, and between feline coronavirus (FCoV) type I and canine coronavirus (CCoV) [43–46]. As shown below, such recombination can result in the generation of coronavirus species or different genotypes within a coronavirus species. In our experience, the possibility of homologous RNA recombination and the possible part of the genome that recombination has taken place are usually first appreciated using bootscan analysis or phylogenetic analysis using different parts of the coronavirus genome. Other methods for recombination analysis, such as those in the RDP3 package, are also available. Then, the exact site of homologous RNA recombination would be best revealed by multiple sequence alignment.

The best documented example of generation of coronavirus species through homologous RNA recombination is the generation of FCoV type II by double recombination between FCoV type I and CCoV. It was first observed that the sequence of the S protein in FCoV type II was closely related to that of CCoV [47,48] but the sequence downstream of the E gene in FCoV type II was more closely related to that of FCoV type I strain than to CCoV [49,50]. This observation suggested that there might have been a homologous RNA recombination event between the genomes of CCoV and FCoV type I, resulting in the generation of FCoV type II. Further analysis by multiple sequence alignments pinpointed the site of recombination to a region in the E gene. A few years later, an additional recombination region in the Pol gene was also discovered, and it was concluded that FCoV type II originated from two recombination events between the genomes of CCoV and FCoV type I [43].

As for the generation of different genotypes in a coronavirus species through homologous RNA recombination, the best documented example is HCoV-HKU1. The possibility of homologous RNA recombination was first suspected when a few strains of HCoV-HKU1 showed differential clustering when the Pol, S and N genes were used for phylogenetic tree construction [51]. This observation has led to our subsequent study on complete genome sequencing of 22 strains of HCoV-HKU1. Recombination analysis by bootscan analysis and phylogenetic analysis using different parts of the 22 complete genomes revealed extensive recombination in different parts of the genomes, resulting in the generation of three genotypes, A, B and C, of HCoV-HKU1 [52]. Using multiple sequence alignment, two sites of recombination were pinpointed. The first one was observed in a stretch of 143 nucleotides near the 3′ end of nsp6, where recombination between HCoV-HKU1 genotype B and genotype C has generated genotype A; and the second one in another stretch of 29 nucleotides near the 3′ end of nsp16, where recombination between HCoV-HKU1 genotype A and genotype B has generated genotype C [52].

## 6. Codon usage bias

Recently, using the complete genome sequences of the 19 coronavirus genomes, we analyzed the codon usage bias in coronaviruses as well as selection of CpG suppressed clones by the immune system and cytosine deamination being the two major independent biochemical and biological selective forces that has shaped such codon usage bias [53]. In the study, we showed that the mean CpG relative abundance in the coronavirus genomes is markedly suppressed [53]. However, we observed that only CpG containing codons in the context of purine-CpG (ACG and GCG), pyrimidine-CpG (UCG and CCG) and CpG-purine (CGA and CGG); but not CpG-pyrimidine (CGU and CGC); are suppressed. However, when trinucleotide frequencies were analyzed in the 19 coronavirus genomes, all the eight trinucleotides with CpG were suppressed [53]. These indicate that another force that has led to an increase use of CGU and CGC as codons for arginine, but does not act on trinucleotides over the whole genome in general, is probably present. Furthermore, this force is probably unrelated to the relative abundance of the corresponding tRNA molecules in the hosts of the coronaviruses, as the pattern of bias in the hosts is not the same as that in the coronaviruses.

In addition to CpG suppression, marked cytosine deamination is also observed in all the 19 coronavirus genomes [53]. Using the six amino acids that are only encoded by NNC or NNU (asparagine, histidine, aspartic acid, tyrosine, cysteine and phenylalanine), hence excluding most other pressures that may affect the relative abundance of cytosine and uracil, it was observed that all NNU are markedly over represented with usage fractions of more than 0.700, whereas the usage fractions of all NNC are less than 0.300 [53]. For all codons that encode the same amino acid and with either U or C in any position, the usage fraction of the codon that uses U is always higher than the one that uses C in all coronaviruses. Furthermore, the percentage of U showed strong inverse relationships with the percentage of C in the coronavirus genomes. These suggest that cytosine deamination is another important biochemical force that shaped coronavirus evolution.

Interestingly, among all the 19 coronaviruses, HCoV-HKU1 showed the most extreme codon usage bias. HCoV-HKU1 is the only coronavirus that had effective number of codons outside the mean ± 2 standard deviations range. In addition, HCoV-HKU1 also possessed the lowest G + C content, highest GC skew, lowest percentages of G and C and highest percentage of U among all coronavirus genomes. Furthermore, HCoV-HKU1 showed extremely high NNU/NNC ratio of 8.835. The underlying mechanism for the extreme codon usage bias, cytosine deamination and G + C content in HCoV-HKU1 is intriguing.

## 7. Database

Rapid and accurate batch sequence retrieval is always the cornerstone and bottleneck for all kinds of comparative genomics and bioinformatics analysis. During the process of batch sequence retrieval for comparative genomics and other bioinformatics analysis of the coronavirus genomes that we have sequenced, we encountered a number of major problems about the coronavirus sequences in GenBank and other coronavirus databases. First, in GenBank, the non-structural proteins encoded by ORF1ab are not annotated. Second, in all databases, the annotations for the non-structural proteins encoded by ORFs downstream to ORF1ab are often confusing because they are not annotated using a standardized system. Third, multiple accession numbers are often present for reference sequences. These problems will often lead to confusion during sequence retrieval. Fourth, coronaviruses, especially SARSr-CoV, amplified from different specimens may contain the same gene or genome sequences, which will lead to redundant work when they are analyzed. In view of these problems, we have developed a comprehensive database, CoVDB, of annotated coronavirus genes and genomes, which offers rapid, efficient and user-friendly batch sequence retrieval and analysis [54]. In CoVDB, first, annotations on all non-structural proteins in the polyprotein encoded by ORF1ab of every single sequence were performed. Second, annotation was performed for the non-structural proteins encoded by ORFs downstream to ORF1ab using a standardized system. Third, all sequences with identical nucleotide sequences were labeled and one can choose to show or not to show strains with identical sequences. Fourth, this database contains not only complete coronavirus genome sequences, but also incomplete genomes and their genes. This is useful because some genes of coronaviruses, such as Pol, S and N, are sequenced much more frequently than others because they are either most conserved or least conserved, and therefore are particularly important for primers design for RT-PCR assays and evolutionary studies.

## 8. Concluding remarks

After the SARS epidemic, there has been a marked increase in the number of coronaviruses discovered and coronavirus genomes being sequenced. This increase in the number of coronavirus species and genomes, a comprehensive and user-friendly database for efficient sequence retrieval, and the ever improving bioinformatics tools have enabled us to perform meaningful genomic, phylogenetic, evolutionary rate and divergence, recombination, and other bioinformatics analyses on the *Coronaviridae* family. Three genera, *Alphacoronavirus*, *Betacoronavirus* and *Gammacoronavirus*, have been used to replace the traditional group 1, 2 and 3 coronaviruses. A fourth genus, *Deltacoronavirus*, which includes BuCoV HKU11, ThCoV HKU12 and MunCoV HKU13, is likely to emerge. Under this new classification system, bat coronaviruses dominate the *Alphacoronavirus* and *Betacoronavirus* genera and bird coronaviruses dominate the *Gammacoronavirus* and *Deltacoronavirus* genera. This huge diversity of coronaviruses in bats and birds has made them excellent gene pools for coronaviruses in these four genera [55].

## Figures and Tables

**Table 1.** Genome comparison of coronaviruses.

Viruses	Hosts	G+C contents	Transcription regulatory sequences	No. of nsp in ORF1ab	No. of papain-like proteases in ORF1ab	No. of small ORFs between ORF1ab and N	Presence of conserved S cleavage site	No. of small ORFs downstream to N
*Alphacoronavirus*								
Transmissible gastroenteritis virus	Pigs	0.38	CUAAAC	16	2	2	N	1
Porcine respiratory coronavirus	Pigs	0.37	CUAAAC	16	2	1	N	1
Feline coronavirus	Cats	0.39	CUAAAC	16	2	4	N	2
Human coronavirus 229E	Humans	0.38	CUAAAC	16	2	2	N	-
Human coronavirus NL63	Humans	0.34	CUAAAC	16	2	1	N	-
Porcine epidemic diarrhea virus	Pigs	0.42	CUAAAC	16	2	1	N	-
Scotophilus bat coronavirus 512	Lesser Asiatic yellow house bats	0.40	CUAAAC	16	2	1	N	1
Rhinolophus bat coronavirus HKU2	Chinese horseshoe bats	0.39	CUAAAC	16	2	1	N	1
Miniopterus bat coronavirus HKU8	Bent-winged bats	0.42	CUAAAC	16	2	1	N	1
Miniopterus bat coronavirus 1A	Bent-winged bats	0.38	CUAAAC	16	2	1	N	-
Miniopterus bat coronavirus 1B	Bent-winged bats	0.39	CUAAAC	16	2	1	N	-
*Betacoronavirus*								
Subgroup A								
Human coronavirus OC43	Humans	0.37	CUAAAC	16	2	1	Y	-
Bovine coronavirus	Cows	0.37	CUAAAC	16	2	3	Y	-
Porcine hemagglutinating encephalomyelitis virus	Pigs	0.37	CUAAAC	16	2	2	Y	-
Equine coronavirus	Horses	0.37	CUAAAC	16	2	2	Y	-
Human coronavirus HKU1	Humans	0.32	CUAAAC	16	2	1	Y	-
Mouse hepatitis virus	Mice	0.42	CUAAAC	16	2	2	Y	-
Subgroup B								
Human SARS related coronavirus	Humans	0.41	ACGAAC	16	1	7	N	-
SARS-related Rhinolophus bat coronavirus HKU3	Chinese horseshoe bats	0.41	ACGAAC	16	1	5	N	-
Subgroup C								
Tylonycteris bat coronavirus HKU4	Lesser bamboo bats	0.38	ACGAAC	16	1	4	N	-
Pipistrellus bat coronavirus HKU5	Japanese pipistrelle bats	0.43	ACGAAC	16	1	4	N	-
Subgroup D								
Rousettus bat coronavirus HKU9	Leschenault's rousette bats	0.41	ACGAAC	16	1	1	N	2
*Gammacoronavirus*								
Infectious bronchitis virus	Chickens	0.38	CUUAACAA	15	1	4	Y	-
Turkey coronavirus	Turkeys	0.38	CUUAACAA	15	1	5	Y	-
Beluga whale coronavirus	Beluga whales	0.39	AAACA	15	1	8	N	-
*Deltacoronavirus*								
Bulbul coronavirus HKU11	Chinese bulbuls	0.39	ACACCA	15	1	1	N	3
Thrush coronavirus HKU12	Gray-backed thrushes	0.38	ACACCA	15	1	1	N	3
Munia coronavirus HKU13	White-rumped munias	0.43	ACACCA	15	1	1	N	3

**Table 2.** Studies on estimation of dates of divergence of SARSr-CoV.

References	Gene	No. of SARSr-CoV strains				Estimated mean substitution rate (no. of substitutions per site per year)	Methods for estimating TMRCA	TMRCA of human/civet SARSr-CoV (95% HPD)	TMRCA of (human/civet)/ Bat Rp3 SARSr-CoV (95% HPD)	TMRCA of (human/civet/Bat Rp3 SARSr-CoV)/SARSr-Rh-BatCoV (95% HPD)
		Human Civet Bat Rp3 SARSr-Rh-Bat CoV								
Zeng *et al.* 2003 [38]	Spike	139				-	Linear regression	Dec 2002 (Sep 2002, Jan 2003)	-	-
Salemi *et al.* 2004 [39]	ORF1ab	10				4/35×10^−4b^	Molecular clock model	-	-	-
Zhao *et al.* 2004 [40]	Genome	16				8–23.8×10^−4^	Three strategies described by the author	Spring 2002	-	-
Song *et al.* 2005 [32]	CDSs^a^	3	5			2.92×10^−3^	Linear regression	Nov 2002	-	-
Vijaykrishna *et al.* 2007 [35]	Helicase	3	3	1	5	2.0×10^−2^, 1.7×10^−2^^c^	Relaxed clock model	1999 (1990–2003)	1986 (1964–2002)	1961 (1918–1995)
Hon *et al.* 2008 [33]	ORF1ab	13	6	1	4	2.79×10^−3^	Various clock models	2002.63 (2002.14–2002.96)	1998.51 (1993.55–2001.32)	∼1985^d^
Lau *et al.* 2010 [37]	ORF1ab	8	8	1	15	2.82×10^−3^	Relaxed clock model	2001 (1999.16–2002.14)	1995.10 (1986.53–2000.13)	1972.39 (1935.28–1990.63)

a Concatenated CDS of ORF1ab, S, E, M and N.

b The rate for all sites is 4×10^−4^. The rate for variable sites is 35×10^−4^.

c Two numbers present the estimated rate of SARSr-Rh-BatCoV lineage and the estimated rate of human/civet/bat SARSr-CoV lineage respectively.

d The date obtained from the figure of the reference but was not mentioned in the reference’s text.
